# Evaluation of African Maize Cultivars for Resistance to Fall Armyworm *Spodoptera frugiperda* (J. E. Smith) (Lepidoptera: Noctuidae) Larvae

**DOI:** 10.3390/plants10020392

**Published:** 2021-02-18

**Authors:** Xavier Chiriboga Morales, Amanuel Tamiru, Islam S. Sobhy, Toby J. A. Bruce, Charles A. O. Midega, Zeyaur Khan

**Affiliations:** 1 International Centre of Insect Physiology and Ecology (icipe), P.O. Box 30772-00100, Nairobi, Kenya; atamiru@icipe.org (A.T.); cmidega@povertyhealth.org (C.A.O.M.); zkhan@icipe.org (Z.K.); 2 Centre for Applied Entomology and Parasitology, School of Life Sciences, Keele University, Keele, Staffordshire ST5 5BG, UK; i.sobhy@keele.ac.uk (I.S.S.); t.j.a.bruce@keele.ac.uk (T.J.A.B.); 3 Department of Plant Protection, Suez Canal University, Ismailia 41552, Egypt; 4 Unit for Environmental Sciences and Management, North-West University, Potchefstroom 2520, South Africa

**Keywords:** fall armyworm, larval feeding, maize, cultivars, host-plant resistance, Africa

## Abstract

The fall armyworm (FAW) has recently invaded and become an important pest of maize in Africa causing yield losses reaching up to a third of maize annual production. The present study evaluated different aspects of resistance of six maize cultivars, cropped by farmers in Kenya, to FAW larvae feeding under laboratory and field conditions. We assessed the arrestment and feeding of FAW neonate larvae in no-choice and choice experiments, development of larvae-pupae, food assimilation under laboratory conditions and plant damage in a field experiment. We did not find complete resistance to FAW feeding in the evaluated maize cultivars, but we detected differences in acceptance and preference when FAW larvae were given a choice between certain cultivars. Moreover, the smallest pupal weight and the lowest growth index were found on ’SC Duma 43′ leaves, which suggests an effect of antibiosis of this maize hybrid against FAW larvae. In contrast, the highest growth index was recorded on ‘Rachar’ and the greatest pupal weight was found on ‘Nyamula’ and ‘Rachar’. The density of trichomes on the leaves of these maize cultivars seems not to be directly related to the preference of neonates for feeding. Plant damage scores were not statistically different between cultivars in the field neither under natural nor artificial infestation. However, plant damage scores in ‘Nyamula’ and ‘Jowi’ tended to be lower in the two last samplings of the season compared to the two initial samplings under artificial infestation. Our study provides insight into FAW larval preferences and performance on some African maize cultivars, showing that there are differences between cultivars in these variables; but high levels of resistance to larvae feeding were not found.

## 1. Introduction

Fall armyworm (FAW) *Spodoptera frugiperda* (J. E. Smith) (Lepidoptera: Noctuidae) is a polyphagous insect pest that had been confined to the tropical and subtropical Americas until it was reported first in Nigeria, Benin, Togo, and Sao Tome and Principe in 2016 [1]. FAW invasion has become the biggest threat to maize production in sub-Saharan Africa due to its rapid spread to almost all countries in the region and associated crop damage [2]. The pest has further spread, during the last two years to India [3], Yemen, Sri Lanka, Bangladesh, Myanmar, Thailand, China [4,5,6], Indonesia, Laos, Malaysia, Vietnam, Egypt, the Republic of Korea, and Japan [7] and the most recent invasions has been detected in Australia, Mauritania, and East Timor [8]. The impact of FAW on maize yield in Africa has been severe [9]. Based on farmers’ perceptions, some authors have estimated the loss of maize yields between 22 and 67% in Ghana and Zambia [2], and between 32% and 47% in Ethiopia and Kenya [10]; resulting in millions of US dollars in losses. Baudron et al. have estimated that, with an incidence of FAW ranging between 32 and 48%, the impact of FAW on maize yield is around 11.5% [9].

Host plant resistance is an important component of integrated pest management [11], thus finding any maize cultivars that are FAW-resistant could be a key aspect for developing sustainable strategies to control this voracious insect and minimize yield losses in a context of low input agriculture in developing countries [12]. Screening for FAW resistant maize germplasm has been carried out exhaustively in the Americas since the 1950s [13]; however, maize germplasm locally adapted to the new geographical distribution of FAW in Africa still requires investigation. Currently, several maize cultivars from CIMMYT collections have been assessed for resistance to FAW in Kenya [14]. However, maize landraces that are the most preferred and are grown by smallholder farmers in Kenya have not been evaluated for resistance to FAW. These open pollinated varieties (OPVs) have been grown by smallholder farmers over generations because they are perceived to be drought and pest tolerant, low costs of acquiring seeds, and give reasonable yields under marginal environmental conditions without application of fertilizers and pesticides [15]. Indeed, studies have reported that they are less affected by the parasitic striga weed, *Striga hermonthica* Benth. (Orobanchaceae) [16], and have superior defense against cereal stemborers [17]. Our study evaluates a number of these OPVs for resistance to feeding by FAW larvae comparing to locally grown hybrids. We hypothesized that FAW larval arrestment (i.e., behavior which makes the insect restrict its movement to a small area), feeding, development and plant damage vary depending on maize cultivar, measures that can be used as a proxy for resistance to FAW. We used laboratory experiments to assess the arrestment, feeding, development and food assimilation of FAW larvae and a field experiment to estimate FAW damage to maize plants. We also measured the density of trichomes on maize leaves as a possible explanation of any variation observed in acceptance and preference by FAW larvae. We discuss our findings in terms of host-plant selection behavior (selection, acceptance, and preference), features of direct resistance of maize cultivars to larvae feeding and the nutritional value of maize cultivars for larvae. The integration of maize resistance with other strategies of sustainable FAW management are also discussed within updated approaches of host-plant resistance [18,19].

## 2. Results

### 2.1. Arrestment/Dispersal and Feeding Experiments in No-Choice Tests

**Figure 1:**
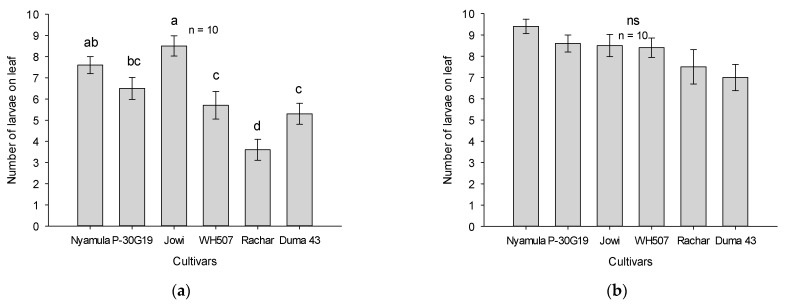
Number of fall armyworm (FAW) larvae (arrestment) on leaf cuts of maize cultivars (average ± SEM; n = 10) in a no-choice experiment after 24 h (**a**) and after 48 h (**b**). Different letters represent significant differences based on Tukey HSD test (5%) and (ns) represents no significant differences.

**Figure 2:**
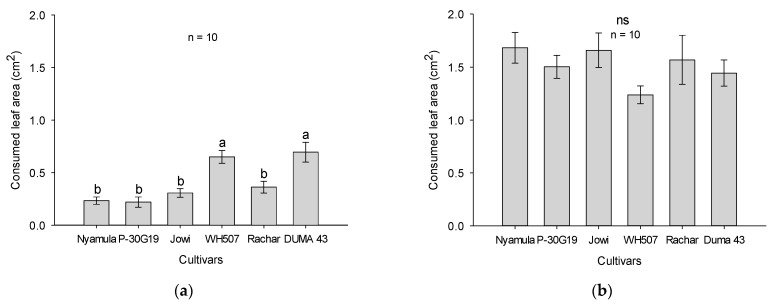
Consumed leaf area (cm^2^; average ± SEM; n = 10) in maize cultivars by FAW neonate larvae in a no-choice experiment after 24 h (**a**) and 48 h (**b**). Different letters represent significant differences based on Tukey HSD test (5%) and (ns) represents no significant differences.

We found significant differences (F_5,54_ = 11.24, *p* < 0.001) in arrestment behavior of FAW neonate larvae on leaf cuts of different maize cultivars after 24 h in no-choice experiments. Larval arrestment was significantly higher in the landrace ‘Jowi’ compared to the hybrids ‘Pioneer 30G19′, ‘WH507′ and ‘SC Duma 43′, and the landrace ’Rachar’ (Figure 1a). However, larvae consumed significantly more leaf area on the hybrids ‘WH507′ and ‘SC Duma 43′ (F_5,54_ = 12.46, *p* < 0.001) than on other cultivars (Figure 2a). In contrast, after 48 h, there were no significant differences between the maize cultivars in terms of arrestment (F_5,54_ = 2.15, *p* > 0.05) and consumed leaf area (F_5,54_ = 1.18, *p* > 0.05) (Figure 1b and Figure 2b).

### 2.2. Orientation/Settling and Feeding in Two-Choice Experiments

**Figure 3:**
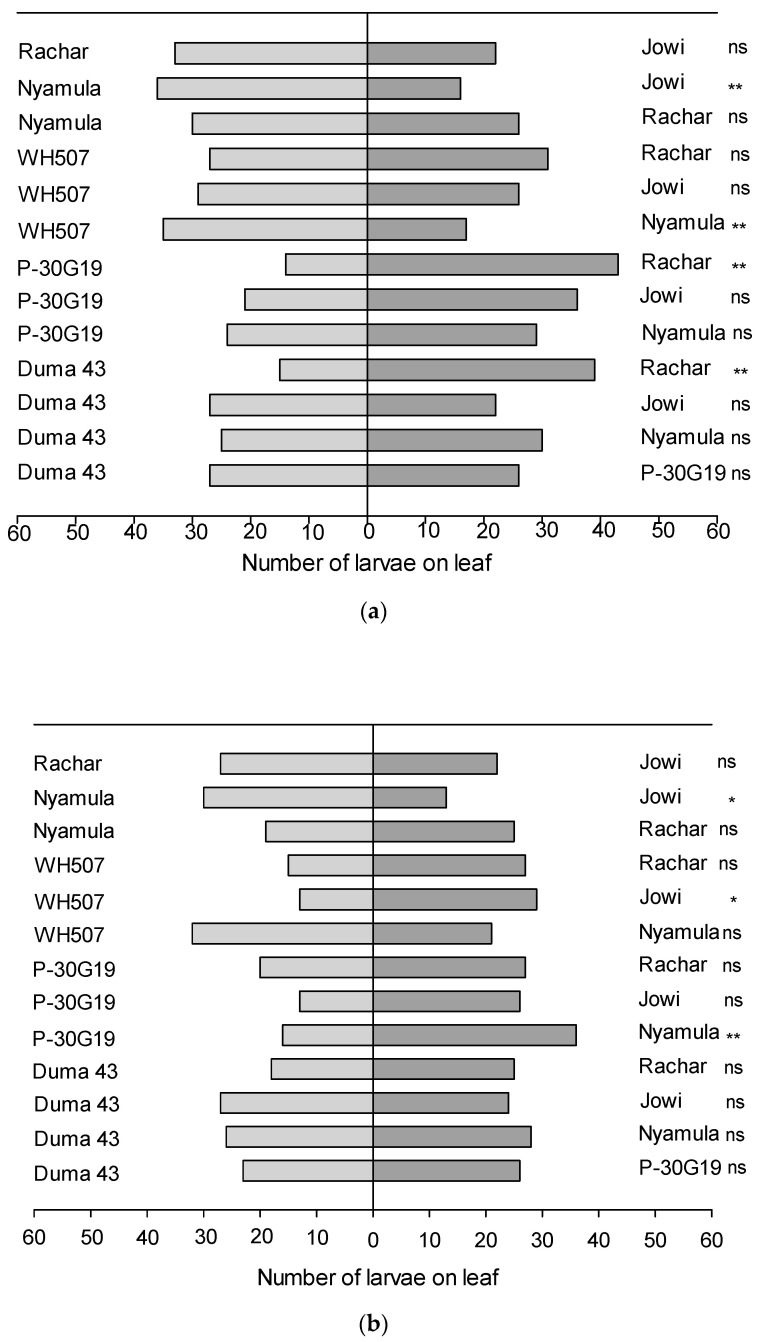
Number of FAW larvae that settled on leaf cuts of different combinations of maize cultivars in a two-choice-experiment (**a**) after 24 h and (**b**) after 48 h. Asterisks represent significant differences (* *p* ≤ 0.05; ** *p* ≤ 0.01) and (ns) represents no significant difference between cultivars (n = 6), based on Chi-square test.

We found significant differences for orientation and settling of FAW neonate larvae on leaf cuts of some choice-combinations of maize cultivars after 24 h. Settling of larvae was significantly higher on the landrace ‘Rachar’ than on the hybrids ‘SC Duma 43′ (*x*^2^ = 9.7, *df* = 1, *p* = 0.001) and ‘Pioneer 30G19′ (*x*^2^ = 13.7, *df* = 1, *p* = 0.0002). On the other hand, significantly less larvae settled on the landrace ‘Nyamula’ than on the hybrid ‘WH507′ (*x*^2^ = 5.5, *df* = 1, *p* = 0.01), and on the landrace ‘Jowi’ than on ‘Nyamula’ (*x*^2^ = 6.9, *df* = 1, *p* = 0.008) (Figure 3a).

We also found significant differences for settling of larvae in some combinations of maize cultivars after 48 h. The number of larvae that settled on ‘Nyamula’ landrace was significantly higher than the number of larvae that settled on the hybrid ‘Pioneer 30G19′ (*x*^2^ = 6.9, *df* = 1, *p* = 0.008), and on the landrace ‘Jowi’ (*x*^2^ = 5.9, *df* = 1, *p* = 0.01). Moreover, significantly higher number of larvae settled on leaf cuts of ‘Jowi’ than on leaf cuts of ‘WH507′ (*x*^2^ = 5.3, *df* = 1, *p* = 0.02) (Figure 3b). The preference for ‘Nyamula’ over ‘Jowi’ was also observed at 24h but other choices varied more between these two time points.

**Figure 4:**
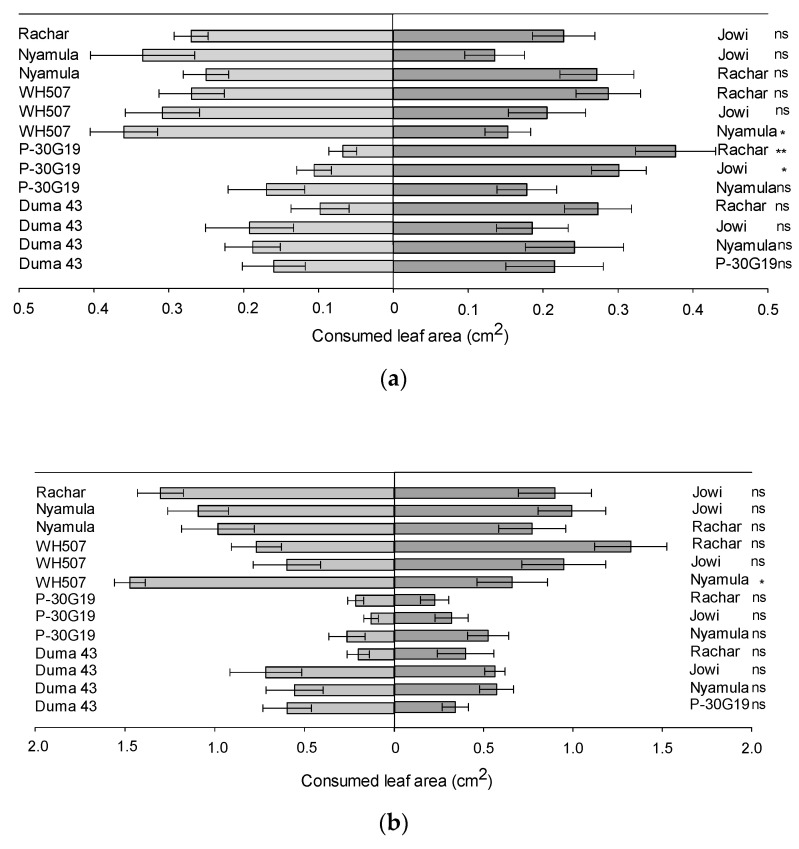
Consumed leaf area (cm^2^; average ± SEM; n = 6) of different combinations of maize cultivars by FAW neonate larvae in a two-choice experiment (**a**) after 24 h and (**b**) after 48 h of feeding. Asterisks represent significant differences (* *p* ≤ 0.05; ** *p* ≤ 0.01) and (ns) represents no significant differences, based on Paired *t*-test.

When assessing feeding after 24 h, we also found differences in consumed leaf area for some combinations. Larvae consumed significantly more leaf area on the landrace ‘Jowi’ than in the hybrid ‘Pioneer 30G19′ (*t* = 3.5, *df* = 5, *p* = 0.01). Similarly, larvae consumed more leaf area on the landrace ‘Rachar’ than on the hybrid ‘Pioneer 30G19′ (*t* = 4.9, *df* = 5, *p* = 0.004), and on the hybrid ‘WH507′ than on the landrace ‘Nyamula’ (*t* = 3.2, *df* = 5, *p* = 0.02) (Figure 4a).

In terms of feeding, after 48 h, a significantly higher leaf consumption was found in the hybrid ‘WH507′ compared to the landrace ‘Nyamula’ (*t* = 3.24, *df* = 5, *p* = 0.02) (Figure 4b).

### 2.3. Larval–Pupal Development Experiment

**Figure 5:**
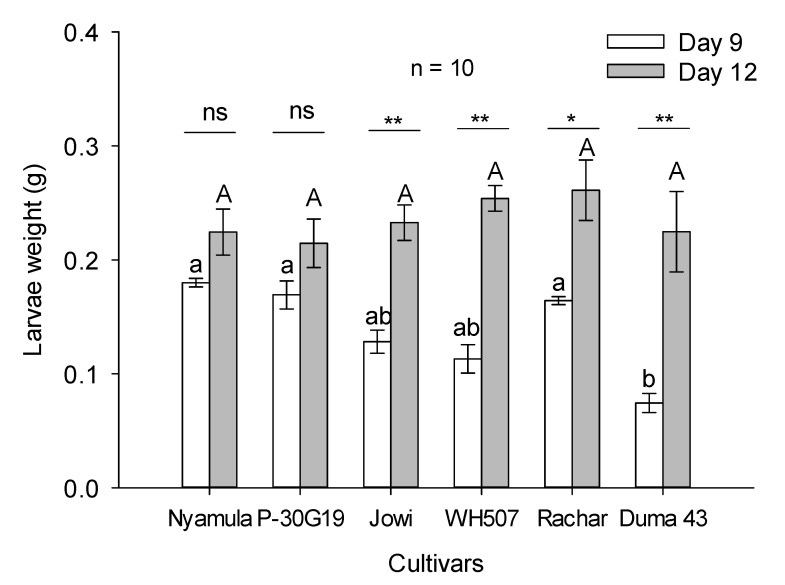
Larval weight (mean ± SEM; n = 10) of FAW developed on different maize cultivars at two time points. Asterisks represent significant differences (* *p* ≤ 0.05; ** *p* ≤ 0.01) between time-points and (ns) represents no significant differences between time-points. Lowercase different letters represent significant differences between maize cultivars within Day 9. Uppercase letters represent no significant differences between cultivars within Day 12, based on Holm–Sidak method (Two-way ANOVA).

We found a significant effect of the maize cultivar (F_5,34_ = 4.46, *p* ≤ 0.05) and developmental time (F_1,34_ = 36.59, *p* ≤ 0.001) on larval weight. The effect of the interaction between cultivar and time (F_5,34_ = 2.65, *p* ≤ 0.05) was also significant. At day nine, larval weight was significantly higher in the maize cultivars ‘Nyamula’, ‘Rachar’, and ‘Pioneer 30G19′ compared to ‘SC Duma 43′. However, we did not find significant differences in the weight of larvae developed on maize cultivars at day twelve (Figure 5).

**Figure 6:**
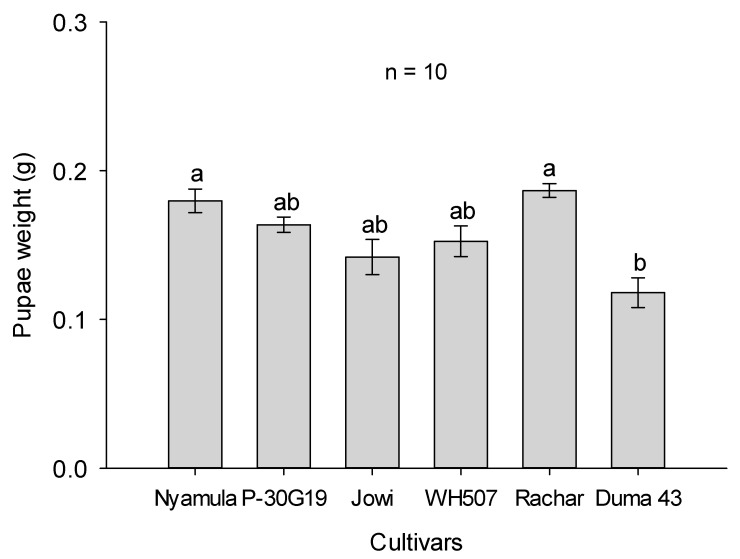
Pupal weight (mean ± SEM; n = 10) of FAW when their larval stage developed on several maize cultivars at day 15. Different letters represent significant differences based on Tukey HSD test (5%).

Furthermore, we found significant differences in the weight of FAW pupae yielded from those larvae developed on different maize cultivars (F_6,27_ = 5.43, *p* ≤ 0.001). Specifically, pupal weight was significantly higher in the landraces ‘Nyamula’ and ‘Rachar’ compared to the hybrid ‘SC Duma 43′ (Figure 6). Differences in pupal weight were similar to differences in larval weight observed at day 9.

The percentage of accumulated mortality was 50% for FAW larvae developed on the landrace ‘Nyamula’ and on the hybrid ‘SC Duma 43′; meanwhile, mortality was 30% and 20% for larvae developed on the landraces ‘Jowi’ and ‘Rachar’, respectively (Table 1). Furthermore, the growth index (calculated as the ratio between percentage of pupation and larval period) was highest for larvae developed on ‘Rachar’ and lowest for larvae developed on ‘SC Duma 43′ (Table 1).

### 2.4. Food Assimilation

The weight of assimilated food was not statistically different (H = 6.51, *df* = 5, *p* = 0.2) between maize cultivars, nor the consumed leaf weight (H = 5.41, *df* = 5, *p* = 0.3). Here, we show the values of both parameters in Table 2.

### 2.5. Leaf Trichome Density

**Figure 7:**
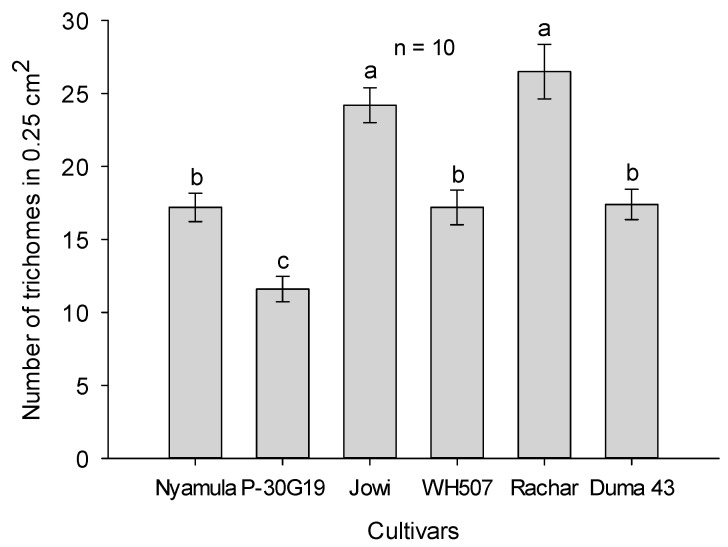
Number of trichomes (mean ± SE; n = 10) in 0.25 cm^2^ of leaves of different maize cultivars. Different letters above bars represent significant differences between cultivars based on Tukey HSD test (5%).

The number of trichomes counted in a leaf area of 0.25 cm^2^ was significantly different (F_5,54_ = 19.37, *p* ≤ 0.001) between maize cultivars. The trichomes density was significantly higher in ‘Jowi’ and ‘Rachar’ compared to the landrace ‘Nyamula’, and the hybrids ‘WH507′, ‘SC Duma 43′ and ‘Pioneer 30G19′ (Figure 7).

### 2.6. Field Experiment

**Figure 8:**
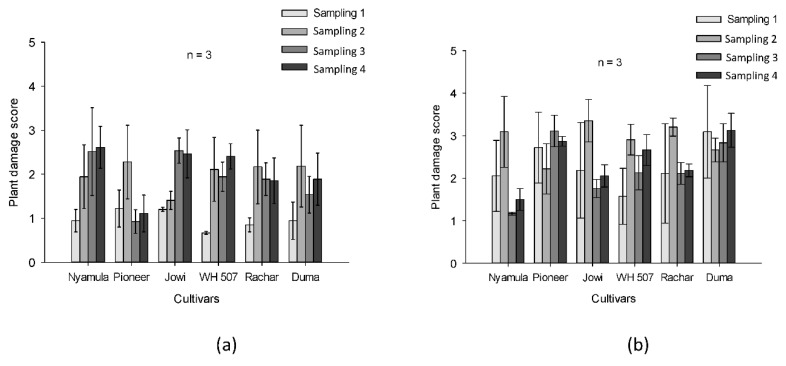
Plant damage scores (mean ± SE, n = 3) for six maize cultivars in cropping conditions across four samplings (sampling 1: 20 days after sowing, sampling 2: 40 days after sowing, sampling 3: 60 days after sowing, sampling 4: 80 days after sowing) in plots: (**a**) naturally infested (**b**) artificially infested.

Overall, we found no significant effect of maize cultivar on plant damage scores in plots under natural infestation (F_5,30_ = 0.78, *p* ≥ 0.05) and artificial infestation (F_5,30_ = 2.17, *p* ≥ 0.05). Plant damage scores were neither affected by sampling-date in plots naturally infested (F_3,30_ = 3.89, *p* ≥ 0.05) nor artificially infested (F_3,30_ = 0.47, *p* ≥ 0.05) (Figure 8). Moreover, the effect of the interaction between cultivar and sampling was not significant under natural infestation (F_15,30_ = 1.15, *p* ≥ 0.05) but was significant under artificial infestation (F_15,30_ = 2.44, *p* ≤ 0.05).

## 3. Discussion

The six maize cultivars in the current study, selected on the basis of being preferred by farmers in the study area, were rigorously evaluated for several aspects of resistance to FAW feeding. None of the cultivars were found to be fully resistant to FAW larvae feeding but some differences in acceptance and preference were observed under choice test conditions. Furthermore, differences in plant suitability of maize leaves of different cultivars were found to impact FAW larval weight and pupal weight. Growth rate on the most favourable cultivars, ‘Nyamula’ and ‘Rachar’, were almost double the growth rate on the least favourable cultivar ‘SC Duma 43′. However, there were no differences between cultivars in leaf weight consumed by larvae under laboratory conditions and in the field experiment no differences were found in plant damage scores.

In Petri-dish experiments, neonate larvae of FAW fed less on ‘Nyamula’ compared to ‘SC Duma 43′ and ‘WH507′ plants after 24 h under no choice conditions; however, no differences were detected between the cultivars after 48 h of experimentation. From the insect point of view, this might be interpreted as subtle differences perceived by the neonate larvae during the test-biting phase; however, these differences may be neglected by the insect as time passes and the need of food increases [20]. In two-choice conditions, FAW neonate larvae arrested less on ‘Nyamula’ compared to the hybrid ‘WH507′ in a period of 24 h, and neonates fed less on ‘Nyamula’ compared to ‘WH507′ after 24 h and 48 h of experimentation. In the choice setups, selection behavior takes place, and we assume that sequential contact-testing of alternative cultivars occurs before a final selection is made by the insect. The results of the two-choice experiment indicate that FAW neonate larvae avoid feeding on ‘Nyamula’ leaf cuts and prefer to feed on ‘WH507′ leaf cuts. There was also a consistent significant preference for settling on ‘Nyamula’ leaf cuts over ‘Jowi’ which was observed both at 24 and 48 h. Since neonates used in our experiments were one-day old, we argue that discrimination is high in these larvae [20]. We discuss possible reasons for these choices below. In our experimental context, acceptance occurs when there is sustained feeding on a leaf cut or plant; meanwhile, preference occurs when an insect, having a choice, consistently feeds more on one of the alternative plant cultivars. The process of host-plant selection and acceptance by an insect involves several behavioral steps and it is a result of the integration of internal physiological state parameters of the insect [21,22]. Initially, when an insect touches a plant, it evaluates physical and chemical plant traits which are often used for an initial behavioral decision, either to proceed or to reject the plant. In the evaluation phase insect restrict their movement to a smaller area, a behavior that is called arrestment. As a next step, the insect may damage the plant by test-biting, when the sensory information is judged positively by its nervous system, the final decision is taken, the host-plant is accepted and food intake starts [22].

Physical features of plant organs or tissues and secondary toxic metabolites can influence host-plant selection behavior and are part of the array of direct defenses of the plant; for example, trichomes, wax crystal structures, leaf thickness and toughness, and silica content may cause avoidance behavior in insects [19,22]. In maize, several characteristics have been reported to confer resistance to maize cultivars against FAW damage. For example, it was found that cuticular lipids in maize leaves play a role in FAW larvae performance. When FAW larvae fed on leaves from which cuticular lipids had been removed, weighed more and developed faster than when they fed on leaves with cuticular lipids [23]. In another study, FAW neonate larvae traveled longer distances and crawled faster on upper leaves, which have a smooth appearance, than on lower leaves, which had a dense array of wax crystals [24]. The presence of trichomes in maize leaves, in our study, did not appear to influence acceptance or preference, since ‘Nyamula’ leaves have a similar density of trichomes to the cultivar ‘WH507′, but are less preferred for feeding. In contrast, trichomes play a defensive role in some rice cultivars against chewing insects [25]. Therefore, we suggest that other physical features such as structure of cuticular compounds, leaf toughness and/or thickness may have been involved in the reduced preference for feeding on ‘Nyamula’ leaf cuts. Another possibility is that antifeedant or repellent chemicals were present on the latter cultivar. On the other hand, lipophilic constituents of leaf surfaces (alkenes, esters, and fatty acids) and secondary plant metabolites are known to promote test-biting and subsequent feeding in many insects [22]. For example, the noctuid *Spodoptera littoralis* Boisduval (Noctuidae), was found to be attracted to nine plant volatiles, proving that plant chemicals play a role in caterpillar searching behavior of host-plants [26]. On the other hand, polyphagous insects species may also be stimulated by the presence of flavonoids in their food [22]. Since acceptance of a host-plant is determined by the balance between stimulatory and inhibitory compounds, a thorough analysis of the compounds in the leaves tissues of these cultivars is needed to unravel their role in host-plant selection and preference.

Our findings regarding food assimilation of tested maize cultivars by third instar larvae were not statistically different. However, there was a trend with larvae consuming relatively more leaf weight of ‘Jowi’ plants compared to ‘Nyamula’, ‘Pioneer 30G19′, ‘Rachar’ and ‘SC Duma 43′. The results also showed relatively less food assimilation when larvae fed on ‘Jowi’ leaves compared to ‘Pioneer 30G19′, ‘WH507′ and ‘Rachar’ leaves. The higher consumption of ‘Jowi’ leaves compared to the other four cultivars suggests that there may be differences in leaf composition. A previous study has demonstrated, for example, that the contents of hemicellulose and cellulose are higher in the whorls of FAW resistant maize plants compared with susceptible plants [27]. Another study has shown the role of leaf toughness in maize resistance to the European corn-borer *Ostrinia nubilalis* Hübner (Crambidae), finding that this character seems to be an important defense mechanism in maize across diverse groups of germplasm [28]. In addition, several factors unrelated to preference influence how much an insect eats; for example, larvae may consume more of a plant low in protein or high in protein inhibitors to compensate for its lower nutritional value [21].

Through experiments investigating insect weight gain, we show that FAW larvae (at day nine) gained more weight on ‘Nyamula’ and ‘Rachar’ leaves compared to the same instars developed on ‘SC Duma 43′ leaves. The same pattern was also observed for the pupal weight at day fifteen. Thus, we argue that ‘SC Duma 43′ leaves are detrimental for FAW larvae development; meanwhile, ‘Nyamula’ and ‘Rachar’ are optimal. Interestingly, larval weight was not different between cultivars at day twelve. This may be explained by the fact that the larvae that grew slower could have attained the same weight of larvae entering the prepupal stage at day twelve. Furthermore, we found differences in growth index between ‘Nyamula’ and ‘Rachar’ that are derived from differences in mortality (see Table 1). Looking together at larval mortality (50%) and low larval-pupal weight when larvae fed ‘SC Duma 43′ leaves, it is clear why growth index for ‘SC Duma 43′ was the lowest (See Table 1). Therefore, we suggest that antibiosis against FAW larvae exist in the cultivar ‘SC Duma 43′. Further investigations need to focus on analyzing factors affecting growth of FAW immature instars such as protein–carbon ratios, allelochemicals, and protease inhibitors [22,29]. Indeed, several investigations have demonstrated the effects of certain maize cultivars on larvae weight [30,31], development time and mortality of FAW larvae [32,33] when used as food. It has been shown that, for example, certain types of flavones-C-glycosides and chlorogenic acid are responsible for antibiosis activity in maize plants [11]. Other mechanisms of defense, such as maize defense genes [34] or toxic proteins [35] are triggered by FAW feeding. Characters of plants’ cultivars that confer them partial resistance to insects often have effects on their biology and further success [19,36].

In terms of insect–plant interactions, the field experiment reflects two different scenarios. In artificially infested plots, FAW larvae were forced to feed on a predetermined cultivar (a no-choice situation) and maize plants were challenged to defend themselves against FAW feeding. Meanwhile, in naturally infested plots, the preference of larvae for feeding (a choice situation) takes place. Indeed, it has been proposed in a model that FAW female moths oviposit indiscriminately on different hosts but neonate larvae leave the natal hosts plants by ballooning, search and colonize suitable hosts [37]. Although we measured plant damage by FAW larvae in the field set-up with a subjective scale, our method is a standard procedure that has been used in FAW resistance research programs for many years [38]. We did not find any significant differences in plant damage scores between maize cultivars under artificial or natural infestation. However, under artificial infestation, which reflects high insect pest pressure, the landraces ‘Nyamula’ and ‘Jowi’ had lower damage scores later in the season which may suggest the potential for compensatory regrowth of these maize cultivars. Indeed, maize plants can display tolerance in response to insect attack [39]. In contrast, these cultivars under natural infestation, showed a slight increase in plant damage scores at the end of the season, which indicate that these cultivars may have borne successive colonization beyond the second sampling of the season. The cultivars ‘WH507′, ‘Rachar’, and ‘SC Duma 43′ showed stable plant damage scores across the three last samplings of the season under natural infestation. Furthermore, plant resistance evaluations in the field become complex due to the interaction of the plant with several other factors, such as location, soil type, plot area, agroecological conditions, weeding frequency, use of hedgerows, intercrops, manure, nitrogen, and year [9,40]. As Ni and his colleagues recommended, environmental conditions and predatory insects abundance should be taken into account when assessing the resistance of maize cultivars to FAW and other insects in the field [40]. Rove beetles, earwigs and spiders are important predatory insects of FAW [41,42]; however, the sampling effort of predatory arthropods in our study was not big enough to draw conclusions on this aspect (Supplementary Tables S1 and S2). Future studies should also investigate the role of indirect defense against FAW in these maize cultivars. Indeed, several studies have shown that certain maize cultivars emit volatile organic compounds (VOCs) which attract parasitoids of their insect pests, after plant damage [43], insect eggs oviposition [17], or even volatiles are produced by plants as constitutive volatiles [44] to defend themselves. In maize plants VOCs are not only important for parasitoids’ attraction but also for predatory insects attraction [45].

## 4. Materials and Methods

### 4.1. Plants

For laboratory experiments, three weeks-old maize plants of six different cultivars (Nyamula, Pioneer 30G19, Jowi, WH507, Rachar, SC Duma 43) obtained from a commercial seed supplier and local Kenyan farmers were used in this study (Hybrids: Luna Agrovet Mbita, Kenya and Landraces: several Kenyan farmers). Plants were grown in plastic pots filled with agricultural soil from the Mbita farm in a screen house (27 ± 2 °C, 65 ± 5% RH, 12L: 12D) at ICIPE-Thomas Odhiambo Campus (ITOC), Mbita Point (Nyanza Province, Kenya). After germination, 3.5 g of fertilizer (Diammonium Phosphate-DAP, Elgon Kenya) was applied in each pot. We used the leaves 6 and 7, counted according to the leaf over arching method (MAFRA’s Guide to Weed Control: Field Crops, 2020) for all the experiments [46], because younger leaves are preferred by *S. frugiperda* for feeding [47].

### 4.2. Insects

FAW neonate larvae (one-day old) were obtained from a FAW colony reared at the ITOC insectary (25 ± 3 °C, 75 ± 5% RH) with an artificial diet (Southland Products Inc., Lake Village, AR, United States). Neonate larvae were used to perform laboratory and field experiments. Third larval instar (six-days old) was used for the food assimilation experiment.

### 4.3. Arrestment-Dispersal and Feeding Experiment 

An arrestment experiment was done to evaluate the acceptance of six maize cultivars by FAW neonate larvae. The experiment was carried-out in no-choice setup. A leaf cut of 6 cm length (approx. 2.5 cm width) was placed in a Petri dish (9 cm) lined with a moistened filter paper. Thereafter, ten neonate larvae were released on the leaf cut, dishes were kept in dark conditions and the number of neonates remaining on the leaf cut was counted after 24 h and 48 h. Ten replicates were used per each maize cultivar. Using the same set-up, we quantified the larval feeding by taking a photo of the leaf cut with a smartphone after the set time. The consumed leaf area fed was then measured automatically using the App Leafbyte (©2018 Zoe Getman-Pickering).

### 4.4. Orientation-Settling and Feeding Experiment 

An orientation and settling experiment was performed in a two-choice setup to evaluate acceptance of FAW neonate larvae to the tested maize cultivars. We used combinations of two hybrids, two landraces or a hybrid with a landrace for setting the choices. Two leaf cuts of 3 cm length (approx. 2.5 cm width) were placed together. The leaf cuts were quadruple shaped and inclined, touching each other by two of their corners, in a Petri dish (9 cm) lined with a moistened filter paper. A total of ten neonate larvae were used in each Petri dish, where five neonate larvae were released up of the touching corners of the leaf cuts and five were released down of the corners, for the larvae to choose a preferred leaf cut. The dishes were kept in dark conditions and number of neonates settled on the leaf cuts was counted after 24 h and 48 h. Six replicates were used per combination. Larval feeding was quantified as mentioned above. Both methods were adapted from Smith et al. [48].

### 4.5. Larval-Pupal Development

A larval development experiment was carried out in laboratory conditions (25 ± 3 °C, 75 ± 5% R.H.) to evaluate the weight gain of FAW larvae that fed on different maize cultivars and thereafter pupal weight. FAW neonates were placed individually in glass vials (2.8 cm × 7.3 cm) with leaf cuts of a maize cultivar. Leaf cuts were obtained from the youngest leaves of plants grown in screen-house conditions and ten replicates were used per maize cultivar. Every 3 days alive larvae were weighed and eaten leaf cuts were replaced by fresh leaf cuts in the glass vials during 15 days. Number of dead larvae and the weight of pupae were also recorded. Here we report the larval weight after 9 and 12 days. We calculated the growth index for FAW larvae fed on the six maize cultivars as the ratio between the percentage of neonate individuals that reach pupal stage and the average time needed for pupation (larval period) [48].

### 4.6. Food Assimilation 

We use the procedure proposed by Smith et al. [48]. FAW larvae (3rd instar) were starved for one hour in a container with moisten paper towel. A leaf cut of 7 cm length was cut off the plant and weighed using a Mettler Toledo PM 460 scale (Mettler Instruments; Greiffensee, Switzerland). Leaf cuts were obtained from new fully open leaves of maize plants of the six cultivars and placed inside glass vials (2.8 cm × 7.3 cm). Ten replicates were performed per cultivar. Glass vials contained a piece of moisten cotton in the bottom. The initial weight of the larvae (W1) was recorded before introducing them into the glass vial, then vials were plugged with cotton and kept in dark conditions. After 24 h of feeding, the weight of larvae (W2) and the remaining leaf cuts were recorded. To estimate the weight loss due to metabolism ten larvae were kept in glass vials with the wet cotton but without leaf cuts, their weight was also recorded at the start (C1) and at the end (C2) of the experiment. Leaf cuts were also kept in vials with the wet cotton but without insects to control their loss of water. The consumed weight of leaf was calculated by subtracting the final leaf weight from the initial leaf weight. The food assimilated (AF) was calculated with this formula:

$$AF=W1\;\left(\frac{C1-C2}{C1}\right)+\left(W2-W1\right)$$

where *AF* is the assimilated food, *W*1 is the initial weight of larvae, *W*2 is the final weight of larvae, *C*1 is the initial weight of unfed larvae and *C*2 is the final weight of unfed larvae.

### 4.7. Leaf Trichome Density

The numbers of trichomes were counted in an area of 0.25 cm^2^ (0.5 cm × 0.5 cm). Samples were obtained from the intermediate position between leaf margin and midrib of the leaf lamina and halfway between proximal end and base of the leaf, from the leaves 6 and 7 of three weeks old plants. Trichomes were counted with a stereo microscope (Stemi 508 Carl Zeiss; Jena, Germany) with a magnification of 25×, moistening slightly the leaf cut [49].

### 4.8. Field Experiment

A field experiment was carried out using a Randomized Complete Block design (RCBD) using three hybrids (SC Duma 43, Pioneer 30G19 and WH507) and three landraces (Jowi, Nyamula and Rachar) of maize (see field layout in Supplementary Figure S1), with three plot-replicates per maize cultivar in two blocks (natural infestation and artificial infestation). Maize seeds were sown at a distance of 30 cm between plants and 75 cm between rows, giving a population of 90 plants per plot. The first data recording was obtained 20 days after the sowing date, and thereafter every twenty days, four times during a season. Plant damage score was recorded, with an adapted visual scale of damage (0–9) used by van Huis [42], evaluating eighteen plants per plot. The experiment was done in the long-rain season (March–May) of 2020. In this experiment, the plots of one block were infested artificially by-hand using a camel-hair brush with ten FAW neonates per plant and the other block sustained a natural infestation by FAW. In the eighteen plants that were evaluated in the field experiments, we counted the number of predators. We report the densities of predatory insects and spiders (Supplementary data) which are known to predate on FAW larvae [42] in each maize cultivar.

### 4.9. Statistics

For no-choice experiments, data of arrestment were square root transformed since data did not have a normal distribution. Then data were analyzed with One-way ANOVA and the means were separated by Tukey HSD test (5%). Data of consumed leaf area were analyzed with One-way ANOVA and means were separated with Tukey HSD test (5%). For two choice experiments, data of settling were analyzed with a Chi-square Goodness of Fit test and data of leaf consumption were analyzed with a paired *t*-test. Data of larvae development were analyzed with Two-ways Repeated Measures ANOVA and pupae development were analyzed with One-way ANOVA. Data of assimilated food weight and consumed leaf weight were analyzed with Kruskai–Wallis test. Data of leaf trichome density were analyzed with One-way ANOVA and means were separated by Tukey HSD test (5%). Plant damage scores from field data were analyzed with Two-way Repeated Measures ANOVA separately for natural and artificial infestation. All data were analyzed, transformed and checked for normality using SigmaPlot 14 (Systat Software Inc., San Jose, CA, USA).

## 5. Conclusions

Taken together these findings suggest that there is no complete resistance to FAW larval feeding in the evaluated maize cultivars. However, there are differences in acceptance and preference between certain maize cultivars. For example, when larvae are given a choice between the landrace ‘Nyamula’ and the hybrid ‘WH507′ they preferred to feed on the latter. The density of trichomes on maize leaves seems not to be associated with preferences of neonate larvae for feeding. The greatest pupal weight was recorded on the leaves of the landraces ‘Nyamula’ and ‘Rachar’; meanwhile, the smallest pupal weight was found on the hybrid ‘SC Duma 43′. Moreover, the lowest growth index was observed on ‘SC Duma 43′. Therefore, our findings suggest that ‘SC Duma 43′ leaves have antibiosis effect against FAW larvae growth. In contrast, the highest growth index was calculated on ‘Rachar’, which indicates this cultivar is more suitable for FAW growth. Plant damage scores were statistically not different between cultivars in the field neither under natural nor artificial infestation. Moreover, scores tended to be lower in ‘Nyamula’ and ‘Jowi’ at the end of the season under artificial infestation. For successful pest management in low input agriculture, it is desirable to combine more than one measure to keep plant damage at low levels. Therefore, it is necessary to study further how other FAW control options, such as biological control and habitat management could be integrated with maize cultivars with partial resistance to FAW. This study contributes to identify cultivars with partial resistance to FAW that can be used with other management options in Africa.

## Figures and Tables

**Table 1:** Mortality, pupation, larval period, and growth index of FAW larvae fed on six maize cultivars.

Maize Cultivar	Percentage of Mortality (%)	Percentage of Pupation (%)	Mean Larval Period (Days)	Growth Index
Nyamula	50	50	15.0	3.33
Pioneer 30G19	30	70	15.0	4.67
Jowi	30	70	15.9	4.41
WH507	40	60	15.0	4.00
Rachar	20	80	15.0	5.33
SC Duma 43	50	50	16.8	2.98

**Table 2:** Assimilated food weight (g) and consumed leaf weight (mean ± SE) by FAW larvae fed on six maize cultivars for 24 h.

Maize Cultivar	Assimilated Food Weight (g)	Consumed Leaf Weight (g)
Nyamula	0.042 ± 0.006	0.08 ± 0.01
Pioneer 30G19	0.050 ± 0.007	0.08 ± 0.01
Jowi	0.037 ± 0.003	0.11 ± 0.01
WH507	0.057 ± 0.011	0.10 ± 0.03
Rachar	0.056 ± 0.006	0.07 ± 0.01
SC Duma 43	0.043 ± 0.004	0.08 ± 0.02

## Supplementary Materials

The following are available online at https://www.mdpi.com/2223-7747/10/2/392/s1, Figure S1: Design of the field experiment in two blocks (natural infestation-normal letters) and artificial infestation-bold letters) with six cultivars and three replicates, Table S1: Densities of insect predators per plant associated with the studied maize cultivars in the field during two seasons, Table S2: Densities of spiders per plant associated with the studied maize cultivars in the field during two seasons.
