# Supplementary material for: Evaluation of African Maize Cultivars for Resistance to Fall Armyworm Spodoptera frugiperda (J. E. Smith) (Lepidoptera: Noctuidae) Larvae
Source: Plants (Basel). 2021 Feb 18;10(2):392. doi: 10.3390/plants10020392 (PMC7922265; doi:10.3390/plants10020392)
Supplement: Supplementary file 1 [file plants-10-00392-s001.zip › supplementary/Resistant genotype to FAW larvae feeding_Sup.Tables_for SUBMISSION_reviewed and corrected.doc]

Table S1. Densities of insect predators per plant associated with the studied maize cultivars in the field during two seasons.

|  | SHORT-RAINS SEASON 2019 (average 6 plots) | | | | LONG-RAINS SEASON 2020^a^ (average 3 plots) | | | | LONG-RAINS SEASON 2020^b^ (average 3 plots) | | | |
| --- | --- | --- | --- | --- | --- | --- | --- | --- | --- | --- | --- | --- |
| Genotype | Sampling  1 | Sampling  2 | Sampling  3 | Sampling  4 | Sampling  1 | Sampling  2 | Sampling  3 | Sampling  4 | Sampling  1 | Sampling  2 | Sampling  3 | Sampling  4 |
| NYAMULA | 0.02 | 0.111 | 0.509 | 0.315 | 0.155 | 0.162 | 0.155 | 0.570 | 0.043 | 0.069 | 0.554 | 0.457 |
| P-30G19 | 0.05 | 0.269 | 0.630 | 0.407 | 0.134 | 0.166 | 0.603 | 0.711 | 0.100 | 0.138 | 0.357 | 0.316 |
| JOWI | 0.009 | 0.167 | 0.463 | 0.454 | 0.173 | 0.278 | 0.224 | 0.498 | 0.030 | 0.144 | 0.511 | 0.557 |
| WH507 | 0.019 | 0.417 | 0.435 | 0.361 | 0.220 | 0.146 | 0.366 | 0.411 | 0.051 | 0.107 | 0.305 | 0.563 |
| RACHAR | 0.009 | 0.148 | 0.481 | 0.167 | 0.131 | 0.270 | 0.336 | 0.876 | 0.078 | 0.135 | 0.571 | 0.310 |
| SC DUMA 43 | 0.028 | 0.130 | 0.269 | 0.120 | 0.171 | 0.209 | 0.226 | 0.519 | 0.110 | 0.201 | 0.174 | 0.223 |

Table S2. Densities of spiders per plant associated with the studied maize cultivars in the field during two seasons.

|  | SHORT-RAINS SEASON 2019 (average 6 plots) | | | | LONG-RAINS SEASON 2020^a^ (average 3 plots) | | | | LONG-RAINS SEASON 2020^b^ (average 3 plots) | | | |
| --- | --- | --- | --- | --- | --- | --- | --- | --- | --- | --- | --- | --- |
| Genotype | Sampling  1 | Sampling  2 | Sampling  3 | Sampling  4 | Sampling  1 | Sampling  2 | Sampling  3 | Sampling  4 | Sampling  1 | Sampling  2 | Sampling  3 | Sampling  4 |
| NYAMULA | 0.09 | 0.204 | 0.398 | 0.380 | 0.047 | 0.065 | 0.040 | 0.083 | 0.022 | 0.022 | 0.043 | 0.051 |
| P-30G19 | 0.06 | 0.167 | 0.546 | 0.593 | 0.058 | 0.047 | 0.047 | 0.105 | 0.015 | 0.026 | 0.074 | 0.063 |
| JOWI | 0.083 | 0.093 | 0.454 | 0.278 | 0.047 | 0.039 | 0.071 | 0.027 | 0.102 | 0.038 | 0.053 | 0.038 |
| WH507 | 0.037 | 0.167 | 0.380 | 0.231 | 0.077 | 0.057 | 0.053 | 0.020 | 0.022 | 0.040 | 0.029 | 0.026 |
| RACHAR | 0.065 | 0.111 | 0.602 | 0.407 | 0.019 | 0.031 | 0.050 | 0.193 | 0.037 | 0.041 | 0.029 | 0.033 |
| SC DUMA 43 | 0.074 | 0.157 | 0.361 | 0.426 | 0.038 | 0.045 | 0.038 | 0.052 | 0.008 | 0.019 | 0.011 | 0.030 |

^a^ Natural and ^b^ Artificial infestation.

Predatory insect species were identified as *Diaperasticus erythrocephala* (Olivier) (Dermaptera: Forficulidae), *Paederus* sp. (Fabricius) (Coleoptera: Staphylinidae), *Cheilomenes vicina* (Muls) (Coleoptera: Coccinellidae), *Epilachna* sp. (Coleoptera: Coccinellidae), whereas spiders were not identified.
