# Supplementary material for: Evaluation of African Maize Cultivars for Resistance to Fall Armyworm Spodoptera frugiperda (J. E. Smith) (Lepidoptera: Noctuidae) Larvae
Source: Plants (Basel). 2021 Feb 18;10(2):392. doi: 10.3390/plants10020392 (PMC7922265; doi:10.3390/plants10020392)
Supplement: Supplementary file 1 [file plants-10-00392-s001.zip › supplementary/Resistant genotypes to FAW larvae feeding_Sup.Figure_for SUBMISSION.doc]

| PIONEER  6 |  | WH 507  5 |  | JOWI  4 |  | DUMA  3 |  | RACHAR  2 |  | NYAMULA  1 |
| --- | --- | --- | --- | --- | --- | --- | --- | --- | --- | --- |
|  |  |  |  |  |  |  |  |  |  |  |
| RACHAR  6 |  | JOWI  5 |  | NYAMULA  4 |  | PIONEER  3 |  | DUMA  2 |  | WH 507  1 |
|  |  |  |  |  |  |  |  |  |  |  |
| DUMA  6 |  | NYAMULA  5 |  | RACHAR  4 |  | WH 507  3 |  | JOWI  2 |  | PIONEER  1  35.2m |
|  |  |  |  |  |  |  |  |  |  |  |
| **WH 507**  **6** |  | **PIONEER**  **5** |  | **DUMA**  **4** |  | **RACHAR**  **3** |  | **NYAMULA**  **2** |  | **JOWI**  **1** |
|  |  |  |  |  |  |  |  |  |  |  |
| **JOWI**  **6** |  | **DUMA**  **5** |  | **PIONEER**  **4** |  | **NYAMULA**  **3** |  | **WH 507**  **2** |  | **RACHAR**  **1** |
|  |  |  |  |  |  |  |  |  |  |  |
| **NYAMULA**  **6** |  | **RACHAR**  **5** |  | **WH 507**  **4** |  | **JOWI**  **3** |  | **PIONEER**  **2** |  | **DUMA**  **1** |

Figure S1. Design of the field experiment in two blocks (natural infestation-normal letters) and artificial infestation-bold letters) with six cultivars and three replicates.

0.75m

2m

0.3m

2m

4.2m

(15 maize plants)

3.75m

32.5m
